# Empyema with Recovery in a Boy Two-and-a-Half Years1Read at the thirty-third annual meeting of the Medical Association of Central New York, held at Rochester, N. Y., October 16, 1900.

**Published:** 1901-03

**Authors:** M. L. Bennett

**Affiliations:** Watkins, N. Y.


### EMPYEMA WITH RECOVERY IN A BOY TWO-AND-A-HALF YEARS.[1]

By M. L. BENNETT, M. D., Watkins, N. Y.

EMPYEMA, or an accumulation of pus in the pleural cavity, is quite a rare disease and is more likely to occur as an inflammatory complication of pneumonia or pleuro-pneumonia, and more often in the child than in the adult. The first symptoms are a sudden rise in temperature, with a distressing cough, great dyspnea, with severe pain, restlessness, the skin bathed in a clammy perspiration, a rapid pulse, the temperature ranging from 102° to 104°; respiration, 40 to 50. There is dulness on the affected side with change of sound on auscultation or percussion when sitting or lying, or if the patient is turned on his side. As the hours pass the affected side becomes more fixed, the intercostal spaces are even, sometimes bulging, the respiratory murmur is *nil*, and the dulness is complete when the chest fills with pus. There is great exhaustion and emaciation from the beginning, with loss of appetite and sleep.

The diagnosis of empyema is not always easy in the early stages, when the pus is in small quantity, and it may be hard to differentiate from some forms of lung trouble, except for the variety of sounds one hears in most all the early stages of pneumonia upon auscultation and percussion, and in empyema the dulness being more complete.

The prognosis of this disease in young children is not very favorable, therefore it should not be left long to itself, and the sooner the pus is removed the better will be the chances of recovery. The danger to life is chiefly due to the complications,—pericarditis, septicemia and rupture into the lung or bronchi,—but besides these complications the most serious is the deformity that occurs in the chest and spine following the collapsing lung.

Now, as to treatment of empyema. In my opinion we can expect but little from medicine in curing our patient. To be sure, it is wise to look well to everything that will in any way add to his comfort and welfare. The alterative doses of mercury act well, also the hypophosphites with a concentrated diet, but you will find that your sheet anchor is in getting rid of the pus early, either by aspiration or incision and the introduction of the drainage-tube. In most cases aspiration should be limited to one or two trials and if that fails, then free incision and drainage. The best point selected for puncture, is

1. Read at the thirty-third annual meeting of the Medical Association of Central New York, held at Rochester, N. Y., October 16, 1900.

the posterior axillary line at the extreme angle of the scapula, between the 6th, 7th or 8th ribs, selecting the most pendant portion of pus.

The method of simple incision, large enough to allow the insertion of the drainage-tube, may prove all that is necessary and is much safer in children than resection of a rib, because the shock is no more than that received from aspirating. This should be followed by irrigation after draining of the pus, either with dioxide or bichloride and then washing out with normal salt. This solution should be warm, leaving some of the normal salt in the cavity.

I will report a case to illustrate:

Walter D., a little boy, two-and-a-half years of age, of healthy family history, was suddenly taken ill with lobar pneumonia of the left side on February 15th of this year. The inflammation commenced in the upper lobe and in a few hours implicated the whole lung. At this time the temperature was 103°; pulse, 150, and respiration 40. The little patient was nervous, so that it was impossible to use cold external applications, and hot poultices were used instead and a syrup of the muriate of ammonia, in teaspoonful doses, was given every two hours; also, we gave a tablet containing calomel, ipecac and soda, as a laxative Under this treatment, the disease ran nearly the usual course, with the temperature ranging from 102° to 104°. On the tenth day the temperature fell to 101° and on the evening of the eleventh day it dropped to the normal, with a corresponding reduction in pulse and respiration. In three days after this, the temperature began to rise, reaching a 102°, the respiration 40, with a harrassing cough; the child restless and considerable pain. All these symptoms I regarded as very suspicious; frequent examinations of the chest were made and in a few days they revealed the fact that we had an effusion in the pleural cavity. Here there was the problem of dealing with a pleurisy or empyema, as a secondary complication of the pneumonia, which, in my opinion, is always secondary and rapid in its occurrence. On the 10th of March, about ten days after, there was found positive evidence of effusion into the pleura. We aspirated, removing about twenty-two ounces of pus; following this there was marked relief in the respirations, and the temperature went down to 101°, and remained at nearly that point about a week. Then we could see that the chest was beginning to refill and continued until it was full. This could be detected from the dulness on percussion and auscultation, also from the slight bulging of the intercostal spaces and the pronounced dyspnea and increasing temperature. So, on April 1st, we again aspirated, removing about the same amount of smooth creamy pus that we removed at the first aspiration. There was the same marked relief to the little patient in the dyspnea, temperature and pulse; the cough got better and the appetite improved, but with all these improvements, there was extreme exhaustion. The little fellow became very much emaciated and the temperature after this vacillated between 100° and 101°; however, he rested better. This state of things continued until May 1st, when we decided to open the chest and put in a drainage-tube, remove the pus and wash out the pleura.

You are aware that it is claimed by some surgeons that cases of empyema will get well by repeated aspirating alone, holding that it is not necessary to institute free drainage. While this may be true, I am doubtful if we are warranted in assuming the risk of contraction of the lung, incident to the accumulation of pus which is likely to be present when such a course is adopted. In this particular case, the accumulation of pus was rapid and, in my opinion, good drainage is the principal thing, as was proved to me in the further care of this case. So, after getting the skin over the point of puncture under the anesthetic effects of cocaine, I opened the chest at the posterior axillary line, between the 8th and 9th ribs, this being selected as the most pendant point for the pus, a No. 8 soft rubber catheter was introduced. After draining off 16 or 18 ozs. of pus, the pleural cavity was washed out with a 25 per cent. solution of peroxide, and this was followed by the introduction of the normal salt solution, leaving a portion in the chest. The tube was left in the chest and the end covered with gauze and cotton, these dressings being removed two or three times a day, or as often as soiled. In a few hours we found that the little patient's temperature was reduced to 99°, the respiration improved, the appetite gradually grew better, and he could sleep through most of the night. In fact, the little patient seemed more comfortable in all respects. The discharge continued to grow less and the collapsed lung began to fill the chest.

The movements were also improved, but as the discharge continued to some extent, we concluded to again wash out the chest, which was done on May 15th. This time mercury bichloride solution, 1 to 4,000 was used, afterward washing out with the normal salt. After this washing the discharges were less frequent and continued to diminish day by day until the middle of June, when they ceased entirely. From this time there has been daily improvement in everything pertaining to the case. The little fellow, who before this illness used to romp and play with the children, had by his severe sickness been reduced to a mere skeleton and was unable to creep or walk; very soon afterward, however, his chest grew better, his strength improved, he soon began to creep, then to walk and play with the children.

The expansion of the lung is fairly good with a little loss of motion below the fifth rib, but he does not seem to favor this side in walking at all. The history of this case is interesting to me, both from the fact that the child was so young and also the length of time from which we commenced the drainage to the time of our removing the tube. One advantage in this method of drainage is that it allows considerable freedom to your patients. They can change their position, and may be removed from the room to another in order to procure pure air.

One of the chief necessities in the management of these cases, is to get the patients in such condition that they can be allowed to get fresh air, for the sooner they can get out the better. Medicinally, this child has taken nothing since recovering from the pneumonia, with the exception of syrup of the hypophosphites and syrup of the iodide of iron. At this writing he is gaining about a pound a week.

This case illustrates fairly, the results of surgical treatment in empyema in a young child, and physicians can readily appreciate that it is much more difficult to care for so young a child than for the adult. In my opinion, the results obtained in this instance were even better than had we performed a resection, as there is no more shock to the patient than by ordinary aspiration. The drainage-tube can be taken out and kept out over night and then returned. This we did several times during the treatment of this child.

Routine treatment in this class of cases is unscientific, as in every other department of medicine, therefore, we must adopt our treatment to the exigencies present in each case. I believe it to be bad judgment to subject every case of empyema to rib section, as it is to disclaim that the necessity of resection ever arises.

### DISCUSSION.

Dr. G. P. Clark, Onondaga—The author said the diagnosis he made was of the fact that he had empyema, and I believe he said that the respirations were *nil*. That, of course, is true in the majority of cases. There is a class of cases, and one often meets with them—at least, I have, a great many times—in which the sounds resemble very closely those of pneumonia, especially in the fact that instead of the respiratory sounds being *nil*, you have very pronounced bronchial breathing. I might say that, to me, the difference between the lobar pneumonia and these cases is that the sound is startling, close to the ear. It seems almost as though it was right under the skin. I have had a chance of proving the diagnosis, I am sorry to say, in a number of cases, by autopsy, and I think it is very well established, that in a good many cases of empyema, you have bronchial breathing instead of an absence of the respiratory sounds.

Dr. Cheesman, of Auburn, once told me that he had met with the condition in grown people upon more than one occasion. I have never seen it except in children, but it is very marked, and it is something that should be kept in mind in diagnosticating those cases. Perhaps it would be well to state that in most of the cases of that kind where I have seen the autopsy, the discharge in the pleural cavity has been very thick indeed, and possibly that accounts to some extent for the bronchial breathing; but if you once hear it, you can never mistake it.

Dr. F. W. LESTER, Seneca.—May I be allowed to ask Dr. BENNETT how many weeks he poulticed that child's side before operation?

Dr. BENNETT.—Only a week or ten days.

Dr. LESTER.—Two cases come to my mind of being called in counsel where there was pus in the pleural cavity. They had been poulticed for a long time. One had been poulticed over six weeks. When I opened the thorax, I drew over three quarts of pus, and there was still another abscess which depressed the heart, and shoved it over to the right side. The one that I opened first was on the left side. I opened it again directly over the heart and drew over a pint from that side. This child's chest, child about eleven years old, had been, as I said, poulticed a long time.

The second case I was called to by this same physician. My incision into the thorax was about an inch and a half long and the pus spurted fully six feet. The stream was larger than my finger and went clear against the wall. In that case, there was curvature of the spine from adhesions following it and great deformity of the body. Of course, this serum that is first diffused into the pleural cavity turns to pus, and by poulticing, the pus increases. I would not dare to say how much was in that second child, aged fourteen, that had this deformity. He lived about six years afterward and took pneumonia on the other side and died. I believe two gallons of pus came out of that child's side. I merely bring to your attention this matter of poulticing. It is well enough to put on a poultice for a few days, but if you continue, you will have trouble.

Dr. R. S. CARR, Wayne.—Speaking of the large quantity of pus, a case comes to my mind, that I thought I would mention at this time. I was called in consultation to see a case of lobar pneumonia in an adjoining village, and it was pure and simple lobar pneumonia. I was called about three weeks later to see the case again and operated as the author has suggested. I brought an ordinary milk-pan as a receptacle for the pus and the milkpan was nearly filled. There were, at least, four and a half or five quarts of pus. There had been no poulticing in that case, simply the pneumonia jacket, that we are all prone to use at this time. So that it is possible for the pus to occur in those large quantities without the use of the poultice.

Dr. LESTER.—The jacket acts as a poultice.

Dr. CARR.—We continue to use the jacket, however; I simply wanted to call attention to that.

Dr. H. B. HAWLEY, Onondaga.—It seems to me the remarks of the two last speakers supplement what was said by Dr. Clark somewhat. The fact that the bronchial breathing so closely resembles pneumonia, leads us to conflict the two. I don't believe that the poulticing or the jacket does any harm! that is, causes the pus to accumulate more rapidly. It is the neglect that does it. You needn't be fooled. A little hypodermic syringe used as an aspirator will, nine times out of ten, clear up those obscure cases. You need not wait six weeks when six hours or six minutes will show you, if you will use your needle, and you are not losing the valuable time that runs your patients down. It is the diagnosis that is at fault.

Dr. N. JACOBSON, Onondaga.—It seems to me this question of empyema is one that must be rewritten altogether. I believe there are a great variety of cases. We have cases of empyema that are due to pneumococcus, some due to pleurococcus and some to the epidermic bacillus, and the course pursued in the different cases depends upon the character of the germs that provoke it. In the cases that are due to pneumococcus, where we have pneumonia infection, it is very possible you may have pneumonia in that class of cases, and you may confound the symptoms, because you have the symptoms of both coexisting. Therefore, it does not follow that your diagnosis of the existence of pus in the pleura is to exclude the diagnosis of a coexisting pneumonia. That class of cases, it has been proven by experience, as far as we have gotten, will do well by aspiration, if any case would at all. Many cases of the pneumococcus empyema clear up after a single aspiration, but I don't say that with the idea of advising aspiration for cases of empyema.

On the other hand, the other forms of germ infection, of which I have spoken, do not clear up after aspiration, and those cases require incision. I do believe this condition of things is more often overlooked than recognised, for I see cases that have gone on for months, often for weeks, and being called on to operate frequently, I have seen these cases that go on to a deformity of the chest, where it requires further procedure to dispose of the trouble that has been produced. I have operated as early as the eight month—one child with empyema, at that age—and I have operated late in life. I have one case in mind, in an adult, where the effusion was allowed to collect until the lower part of the diaphragm rested on a level with the crest of the ilium. There is no excuse, as one speaker has said, for delaying the diagnosis, because your aspirator syringe will tell you; and I believe, as soon as you have pus, you had better evacuate it. It is a question, of course, as to the choice of methods; but a little care, care not to turn the child on the other side and press the heart, and careful incision, makes the operation a very simple one.

As far as poulticing is concerned, I must confess that I have not used a poultice for any purpose whatsoever in years. I think a poultice is a thing we ought to discard for any and every purpose whatsoever; not only for pneumonia, but for everything. I feel that while it may not have anything at all to do with the collection, it is one of the things there is no occasion to use.

Dr. R. C. McLennan, Onondaga—It is a matter of not very large practical application, a matter of scientific interest, but the *x* rays show these effusions in the chest cavity very clearly; and in a case where one can get an *x*-ray machine, this matter of effusion can be demonstrated very conclusively.

